# Effects of contextualized emotional conflict control on domain-general conflict control: fMRI evidence of neural network reconfiguration

**DOI:** 10.1093/scan/nsae001

**Published:** 2024-01-04

**Authors:** Tingting Guo, Xiyuan Wang, Junjie Wu, W. John Schwieter, Huanhuan Liu

**Affiliations:** Research Center of Brain and Cognitive Neuroscience, Liaoning Normal University, Dalian 116029, China; Key Laboratory of Brain and Cognitive Neuroscience, Dalian, Liaoning Province 116029, China; Research Center of Brain and Cognitive Neuroscience, Liaoning Normal University, Dalian 116029, China; Key Laboratory of Brain and Cognitive Neuroscience, Dalian, Liaoning Province 116029, China; Key Research Base of Humanities and Social Sciences of the Ministry of Education, Tianjin Normal University, Tianjin 300382, China; Language Acquisition, Multilingualism, and Cognition Laboratory/Bilingualism Matters, Wilfrid Laurier University, Waterloo N2L3C5, Canada; Department of Linguistics and Languages, McMaster University, Hamilton L8S4L8, Canada; Research Center of Brain and Cognitive Neuroscience, Liaoning Normal University, Dalian 116029, China; Key Laboratory of Brain and Cognitive Neuroscience, Dalian, Liaoning Province 116029, China

**Keywords:** emotional conflict control, domain-general conflict control, fMRI, euSEM, effective connectivity

## Abstract

Domain-general conflict control refers to the cognitive process in which individuals suppress task-irrelevant information and extract task-relevant information. It supports both effective implementation of cognitive conflict control and emotional conflict control. The present study employed functional magnetic resonance imaging and adopted an emotional valence conflict task and the arrow version of the flanker task to induce contextualized emotional conflicts and cognitive conflicts, respectively. The results from the conjunction analysis showed that the multitasking-related activity in the pre-supplementary motor area, bilateral dorsal premotor cortices, the left posterior intraparietal sulcus (IPS), the left anterior IPS and the right inferior occipital gyrus represents common subprocesses for emotional and cognitive conflict control, either in parallel or in close succession. These brain regions were used as nodes in the domain-general conflict control network. The results from the analyses on the brain network connectivity patterns revealed that emotional conflict control reconfigures the domain-general conflict control network in a connective way as evidenced by different communication and stronger connectivity among the domain-general conflict control network. Together, these findings offer the first empirical-based elaboration on the brain network underpinning emotional conflict control and how it reconfigures the domain-general conflict control network in interactive ways.

## Introduction

Processes of suppressing/controlling conflicts, whether cognitive or emotional, are typical in communication and the ability to effectively control conflict helps humans to better adapt to the environment around them. The impairment of emotional conflict control particularly has been manifested in a variety of psychiatric disorders (Wang *et al*., 2008; Etkin *et al*., 2010). Exploring the neural basis of cognitive conflict control and emotional conflict control is of great significance to explain these psychological processes and can have important implications for patients in clinical settings.

Domain-general conflict control refers to a common subprocess which implements efficient performance of cognitive conflict control (Ridderinkhof *et al*., 2004; Gilbert and Burgess, 2008) and emotional conflict control (Etkin *et al*., 2006). It is a process by which individuals extract task-relevant information and suppress task-irrelevant information when confronted with conflicting input. There has been a surge of research examining the neural overlap between emotional conflict control and cognitive conflict control (Torres-Quesada *et al*., 2014; Chen *et al*., 2018), and it is reported that different types of conflict control recruit a domain-general conflict control network in which each brain region plays its own role (Ochsner *et al*., 2009; Xu *et al*., 2016). For example, the pre-supplementary motor area (pre-SMA) is a critical brain region involved in control across emotional and cognitive conflict control (Xu *et al*., 2016; Xing *et al*., 2023). This region is also thought to be associated with conflict monitoring, in which signals are sent to other regions when a conflict is identified (Garavan *et al*., 2003; Iannaccone *et al*., 2015).

It has been known for some time that coordinated fluctuations between specialist regions of the brain are critical for behavior (Friston, 1994) and that the brain is an inherently dynamic organ, capable of flexible reconfiguration in the face of external complexities (Shine and Poldrack, 2018). One manifestation of this reconfiguration is that the pattern of coordinated activities among several brain regions that are similarly activated in different tasks may also be distinct (Hillary *et al*., 2011; Yang *et al*., 2015; Wu *et al*., 2019). For instance, a neuroimaging study by Wu *et al*. (2019) examined the neural correlates of language control and cognitive control and found that language control recruited more connections from frontal to subcortical areas compared with general cognitive control, demonstrating a reconfigurable brain network.

However, previous studies exploring the neural mechanisms underlying cognitive and emotional conflict control have typically been limited to the relative activation between conditions. Most research has not directly tested the patterns of interaction between various key brain regions and has failed to recognize that emotional conflict control may have a reconfiguring effect on the domain-general conflict control brain network. Thus, it is unclear how different types of conflict modulate patterns of functional connectivity between brain areas to support different conflict controls in the domain-general conflict control brain network, which is co-activated by cognitive conflict control and emotional conflict control or whether there is reconfiguration of brain networks for domain-general conflict control that are achieved in connective ways.

### Present study

We administered two tasks involving cognitive and emotional conflict control to a group of individuals. During the tasks, we used functional magnetic resonance imaging (fMRI) to examine the participants’ brain activities. For the cognitive task, we adopted an arrow version of the flanker task which required participants to respond to the direction of the middle arrow while ignoring flanking arrows (see Cognitive Conflict Task section for details). We designed an emotional conflict task to be a contextualized paradigm in which emotional conflicts were triggered by emotions themselves. In the task, participants were required to judge the emotional valence or the opposite emotional valence of words as indicated by an accompanying cue (see Emotional Conflict Task section for details). In choosing the two tasks, our aim was to examine the neural overlap between the two conflict tasks in order to define the domain-general conflict control network for subsequent analyses.

To model the causal interactions (i.e. effective connectivity) of brain regions for cognitive functions, we adopt extended unified structural equation modeling (euSEM; Gates *et al*., 2011). The euSEM method has been widely used in several studies (e.g. Hillary *et al*., 2011; Yang *et al*., 2015; Younger *et al*., 2017; Wu *et al*., 2019) and is a flexible and efficient way of analyzing data based on SEM. The method also allows for exploratory data analyses without prior theoretical assumptions.

We first conducted activation analyses to determine the precise brain regions that were critical for emotional and cognitive conflict control. We then extracted and modeled the time course of each critical region using euSEM for both tasks and obtained connectivity maps for emotional and cognitive conflict control. Wilcoxon signed-rank tests were performed to examine differences in the strength of shared connections for the two types of conflict control. These analyses were then used to examine the specific connectivity patterns of two conflict controls. We hypothesize that the connectivity pattern will reveal that emotional conflict control reconfigures the domain-general conflict control network in connective ways.

## Method

### Participants

Thirty participants were recruited from Liaoning Normal University in China, and three of them were excluded due to poor performance in the two experimental tasks (accuracy <70%). Thus, the participants in the data analyses included 27 individuals (14 females, 13 males, *M* = 21.93 ± 2.35 years), exceeding the minimum sample size of 24 calculated by G*power 3.1.9.7. Parameters were set as follows: F-tests > analysis of variance (ANOVA): repeated measures, within factors, medium effect size *f* = 0.25,^1^ α error probability = 0.05, correlation among repeat measures = 0.5, power (1 − β error probability) = 0.8, number of groups = 2, number of measurements = 4, and nonsphericity correct ∈ = 1. All participants were right-handed, had normal or corrected-to-normal vision and had no history of neurological or psychological disorders. Participants were required to sign an informed consent form before taking part in the study and were given a modest monetary remuneration after their participation. The study was approved by the Ethics Review Committee at Liaoning Normal University.

### Materials and procedure

The experimental procedure involved two tasks: the arrow version of the flanker task, which measured cognitive conflict control, and an emotional valence conflict task, which measured emotional conflict control. The order of the two tasks was counterbalanced across participants. Participants completed practice trials prior to beginning the formal experiment at which time, they proceeded to a separate fMRI laboratory where the testing took place.

#### Cognitive conflict task

**Fig. 1.**
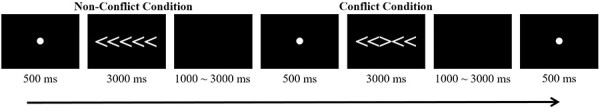
Procedure of the flanker task.

*Note*: Each trial comprised a 500 ms fixation point, followed by the presentation of a stimulus lasting up to 3000 ms and then a random blank screen duration ranging from 1000 ms to 3000 ms. During the presentation of the stimulus, participants were instructed to respond as quickly and accurately as possible to indicate the direction of the middle arrow. Upon the participant’s button press, the stimulus disappeared, and a blank screen was presented to maintain the 3000 ms duration.

The flanker task (Gibson, 1969; Eriksen and Eriksen, 1974) is a classic experiment thought to induce and measure cognitive conflict control (Zavala *et al*., 2013; Imburgio *et al*., 2020). We administered an arrow version of flanker task in which each trial started with a white fixation point (•) on a black background for 500 ms (see Figure 1). The fixation point then disappeared, and five arrows were displayed. Upon seeing the arrows, participants responded to the direction of the middle arrow by pressing the appropriate button on the fMRI keyboard as quickly and accurately as possible (i.e. pressing ‘2’ with their left thumb to answer ‘left’ and ‘3’ with their right thumb to answer ‘right’). The arrows were displayed for 3000 ms or until participants responded, at which time, the screen went blank until the completion of the 3000 ms. Another blank screen randomly appeared for 1000 ms to 3000 ms before the next trial began. Half of the trials were congruent (e.g. <<<<< or >>>>>) and thus represented non-conflict conditions, and the other half were incongruent (e.g. <<><< or >><>>), representing conflict conditions. The order of the trials was randomly presented for each participant. There were 3 warm-up trials and 72 experimental trials, including 36 non-conflict trials and 36 conflict trials. The number of trials in the flanker task was set to match the number of trials in the emotional conflict task (i.e. 36 non-conflict trials of positive words and 36 conflict trials of positive words, specifically mentioned in Emotional Conflict Task section), which were ultimately included in the analysis. The entire task lasted for 7 min.

#### Emotional conflict task

Emotional conflict control typically has been studied in previous research using an emotional face-word Stroop task in which participants make emotional valence judgments about faces while ignoring the valence of words or vice versa (Etkin *et al*., 2006; Rey *et al*., 2014; Jamieson *et al*., 2023). Unlike the classic paradigm which only involves dimension ignoring, we designed an emotional conflict task involving valence processing to induce more contextualized emotional conflicts. In the task, participants are required to judge the emotional valence or the opposite emotional valence of words as indicated by an accompanying cue.

In the task, participants saw individually presented Chinese words that were accompanied by either a + or −. The + symbol indicated a non-conflict situation in which participants were to provide the same emotional valence of the word by pressing the appropriate button on the fMRI keyboard (i.e. pressing ‘2’ with their left thumb to answer ‘positive’ and ‘3’ with their right thumb to answer ‘negative’). The − symbol represented a conflict condition where participants were to respond with the opposite emotional valence. This conflict condition induces a certain level of emotional arousal conflict, which arises from the disparity between words participants saw and the valence with which they needed to respond. Response keys were counterbalanced across participants. Word materials consisted of 18 positive words, 18 negative words and 36 neutral words^2^ (for word list see Supplementary Material). Moreover, ‘+ positive words’ represented a pure non-conflict condition, while ‘− positive words’ cause both stimulus and response conflict that was in line with the incongruent condition of the flanker task. While ‘− negative words’ cause response conflict but no stimulus conflict, and ‘+ negative words’ pairs cause stimulus conflict but no response conflict. Hence, the conflict effect of positive words was only compared with that of the flanker task. Therefore, only positive words were included in the data analyses, while negative words and neutral words were not relevant to the goal of the current study and were therefore considered control trials and did not form part of the analyses.

**Fig. 2.**
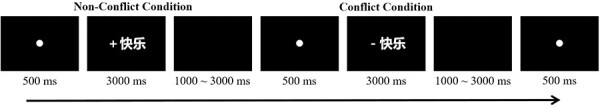
Procedure of the emotional conflict task.

*Note*: The example word (i.e. the Chinese word ‘快乐’) means ‘happy’. Each trial comprised a 500 ms fixation point, followed by the presentation of a stimulus lasting up to 3000 ms, and then a random blank screen duration ranging from 1000 ms to 3000 ms. During the presentation of the stimulus, participants were instructed to respond to the valence of the presented words as quickly and accurately as possible. Upon the participant’s button press, the stimulus disappeared, and a blank screen was presented to maintain the 3000 ms duration.

The procedure of the emotional conflict task is shown in Figure 2. Each trial started with a fixation point (•) on a black background screen for 500 ms, followed by the visual presentation of a target word. The word was displayed for 3000 ms or until participants responded, at which time, the screen went blank until the completion of the 3000 ms. Another blank screen randomly appeared for 1000 ms to 3000 ms before the next trial began. A fixation point then appeared which indicated the start of a new trial. Prior to the formal experiment, participants familiarized themselves with the words’ valence through a reading list of the words and performed a practice set of trials. The procedure of the practice experiment was the same as the formal experiment, but an additional feedback screen was presented after participants’ response to indicate whether their response was correct or not. This feedback screen was intended to assist them in better understanding the task rules and was not present during the formal experiment. The practice experiment consisted of 16 trials, and these words were not included in the formal experiment. After completing the practice experiments, all participants reported that they had mastered the experimental procedure and the task rules.

For the selection of word materials, we asked a separate age-matched group of 24 Chinese speakers to judge their familiarity with 182 Chinese words and their emotional arousal. Both ratings were provided based on five-point scales: for lexical familiarity, ‘1 = very unfamiliar, ‘5’ = very familiar; for emotional arousal, ‘1’ = very mild, ‘5’ = very strong. The ratings showed that their familiarity with the words was not distinct from one another: positive words (*M* = 4.89, *SD* ± 0.16) and corresponding neutral words (*M* = 4.83, *SD* ± 0.14), *t*(1,17) = 1.220, *P* = 0.238; negative words (*M* = 4.80, *SD* ± 0.17) and corresponding neutral words (*M* = 4.78, *SD* ± 0.09), *t*(1,17) = 0.487, *P* = 0.638; positive words and negative words, *t*(1,17) = 1.424, *P* = 0.172. The analyses of emotional arousal demonstrated significant differences between positive words (*M* = 4.42, *SD* ± 0.22) and corresponding neutral words (*M* = 2.18 *SD* ± 0.32), *t*(1,17) = 24.179, *P* < 0.001, and between negative words (*M* = 4.43, *SD* ± 0.20) and corresponding neutral words (*M* = 2.08, *SD* ± 0.28), *t*(1,17) = 29.822, *P* < 0.001. There were no significant differences found between positive words and negative words, *t*(1,17) = 0.132, *p* = 0.896. Based on the analyses of lexical familiarity and emotional arousal, we removed 110 words, leaving 72 experimental words which included 18 positive words (e.g. ‘快乐’, ‘happy’) and 18 negative words (e.g. ‘悲伤’, ‘sad’); and 36 neutral words. These 36 neutral words consist of 18 pairs with opposite meanings at the semantic level, such as ‘big’ and ‘small’, ‘long’ and ‘short’. Among them, ‘big’ and ‘long’ were artificially categorized as ‘positive words’, while ‘small’ and ‘short’ were categorized as ‘negative words’. It is essential to note that this ‘positive/negative’ was different from the ‘positive/negative’ of the emotional words and could be understood as analogous to positive numbers and negative numbers in mathematics. Additionally, the lexical category of Chinese words was not fixed. For instance, 快乐 (happy) could be treated as a noun, adjective or verb. The task was divided into 4 equal blocks, each containing 3 practice and 72 experimental trials. Each block took ∼ min to complete. In total, there were 288 trials: 36 non-conflict trials of positive words and 36 conflict trials of positive words were included in the analyses, and the remaining 72 trials of negative words and 144 trials of neutral words were used as control trials and were not included in the analyses. Trial order was randomized across participants.

### Data acquisition, preprocessing and analyses

#### Data acquisition

Whole-brain image data were acquired by a GE Discovery MR750 3 T scanner. During scanning, participants were laid down with their heads secured with sponges to minimize the head motion. Functional scans were obtained using a T2*-weighted gradient echo planar imaging (EPI) sequence. The following scan parameters for functional images were used: slice thickness = 2 mm, sequential acquisition = 33 axial slices, repetition time (TR) = 2000 ms, echo time (TE) = 30 ms, flip angle = 90°, image matrix = 64 × 64, field of view (FOV) = 224 × 224 mm and voxel size = 3.5 × 3.5 × 4.2 mm. Each functional scanning session contained 215 time points with 5 sessions. Structural images were collected using a three dimensional T1-weighted magnetization prepared rapid gradient echo sequence to co-register with the functional images using the following parameters: TR = 6.652 ms, TE = 2.928 ms, flip angle = 12°, sequential acquisition = 192 slices, slice thickness = 1 mm, spacing between slices = 1 mm, image matrix = 256 × 256, FOV = 256 × 256 mm and voxel size = 1 × 1 × 1 mm.

#### Data preprocessing

Preprocessing of the fMRI data was performed by Data Processing & Analysis for Brain Imaging (Yan *et al*., 2016). All of the EPI Digital Imaging and Communications in Medicine data were converted to Neuroimaging Informatics Technology Initiative format, and the first five volumes of each session were discarded because of T1 relaxation artifacts. Second, slice timing correction was performed for the remaining images using the middle slice in time as the reference slice and the images were realigned to the first volume for head motion correction. Third, the structural images of each participant were co-registered with mean functional images and normalized to the Montreal Neurological Institute template. Fourth, all voxels were resampled to 3 mm cubic voxels. Fifth, all functional volumes were spatially smoothed using an isotropic Gaussian kernel with a 6 mm full width at half maximum.

#### Behavioral and neuroimaging data analyses

The accuracy and reaction times of each participant were analyzed using a two-way repeated measure ANOVA in Statistical Package for the Social Sciences 24. For the RT results, we removed incorrect responses, standardized the RT values (e.g. *z*-scores) and sequentially removed trials that differed by more than ±2.5 SD from their mean RT for each participant. In doing so, this excluded 4.1% of the total data.

Brain imaging data were separately analyzed for the emotional conflict task and cognitive conflict task using Statistical Parametric Mapping (version 8; Wellcome Department of Cognitive Neurology, London, UK, http://www.fil.ion.ucl.ac.uk/spm) in MATLAB. For each participant, a general linear model was used to estimate the effects of the experimental conditions (i.e. conflict and non-conflict) at the voxel-based level, with a reference delta function of stimuli, which was convolved with a canonical hemodynamic response function. Erroneous trials were modeled together as a regressor of no interest and were excluded from the analyses. The data were high-pass-filtered at 128 Hz. At the individual level, we defined the contrast of conflict condition *vs* non-conflict condition in each task with the conflict effect (Kanske and Kotz, 2011; Zinchenko *et al*., 2015). Then, for each task, a one-sample *t*-test was performed on all contrast images to obtain brain activations at the group level.

To construct a network with the same brain regions for emotional conflict control and cognitive conflict control [i.e. to identify regions of interest (ROI) of the domain-general conflict control brain network], a conjunction analysis (conjunction null hypothesis, i.e. only voxels were reported as active if they were significant for the conflict *vs* non-conflict conditions in both tasks) was performed at the group level with a family-wise error (FWE)–corrected threshold of *P* < 0.05 to reject the voxels that were not activated in either of the two tasks (Friston *et al*., 2005; Nichols *et al*., 2005). The average time series of all the voxels in each ROI was extracted by the Resting-State fMRI Data Analysis Toolkit plus (Jia *et al*., 2019) and then used in the subsequent effective connectivity analysis (see Effective Connectivity Analyses section).

In addition, the regions activated by conflict effects of emotional conflict task and flanker task were shown in Tables S1 and S2 (see Supplementary Material), respectively.

#### Effective connectivity analyses

SEM and dynamic causal modeling (DCM) are commonly used to construct the size and direction of the interaction between two brain regions. Among them, DCM examines changes in node activities, which is the sum of direct and indirect changes in node activities caused by external stimuli and a method of model matching data. As such, if there are too many ROIs, the research difficulty will be greatly increased. Considering the number of ROIs in this study, SEM was chosen. SEM defines the connection direction, establishes the model reflecting the variable relationship and then obtains the best-fitting model by adjusting the connection strength. The euSEM is a novel analysis based on SEM. Traditionally, SEM provides a model of the contemporaneous relationships between ROIs and assumes that the observations are independent. However, this is not the case for fMRI time series since the measured fMRI signals are temporally correlated. To address this shortcoming, Kim *et al*. (2007) developed the uSEM that combines lagged (sequentially) relationships with the contemporaneous relationship of a conventional SEM via a multivariate autoregressive model. This analytical approach works well on fMRI data from blocked designs (Kim *et al*., 2007). The euSEM builds on the uSEM and further considers task and bilinear effects (i.e. how the relationship between two nodes changes in the presence of the task) to accommodate data from event-related fMRI studies (Hillary *et al*., 2011; Gates and Molenaar, 2012; Yang *et al*., 2015).

The procedure for using the euSEM is similar to that of Wu *et al*. (2019). To investigate how brain networks are involved in emotional conflict control or cognitive conflict control, euSEM assessed the connectivity patterns of ROIs (which can be represented by a connectivity matrix or map) in the two tasks separately. Using the Group Iterative Multiple Model Estimation (GIMME; Gates and Molenaar, 2012), the selection of euSEM models can be done automatically, which has been shown to outperform other methods that attempt to model the presence of directed connections among nodes at the group and individual levels. We implemented GIMME through the following steps. The model selection procedure began with the use of Lagrange multiplier equivalents (i.e. modification indices; Sörbom, 1989) to determine which effects (including connections among ROIs and for the euSEM and also the direct and bilinear experimental onset terms), if free, optimize the improved model to fit most individuals (more than 75%^3^). Next, the model was pruned by eliminating connections that were no longer significant for 75% of the group after the release of the connections. Then, these connections at the group level were freed and estimated in a semi-confirmatory manner at the individual level. Finally, the confirmatory model was fitted by eliminating individual-level connections that became nonsignificant after releasing other individual-level connections, and the model was pruned. Model fit parameters found to demonstrate that reliability were chosen a priori, so that four criteria were satisfied in the final model: confirmatory fit index (CFI) >0.90; nonnormed fit index (NNFI) >0.90; standardized root mean square residual (SRMR) <0.05; and root mean square error of approximation (RMSEA) <0.08 (Gates *et al*., 2011. The distinctions of the connectivity patterns were examined by a permutation test.

#### Connection strength and hubs

The connectivity maps for emotional and cognitive conflict control have shared connections. Considering the existence of outliers, we used the Wilcoxon sign-rank test, a non-parametric approach which permitted us to check whether the strength of these shared connections differed between the two tasks.

In addition to the connection from one region to another, it is also important to identify the hubs (i.e. detected cores) of the networks. The optimal core–periphery subdivision algorithm (Rubinov *et al*., 2015; Wu *et al*., 2019; Yuan *et al*., 2021), which divides the network into a core group and a periphery group in such a way that the number of edges within the core group is maximized and the number of edges within the periphery group is minimized, was used to determine the hubs. The coreness (*Q*) is reported to quantify the merit of the optimal core–periphery subdivision. The Brain Connectivity Toolbox (Rubinov and Sporns, 2010) was used for the detection of core–periphery structure.

## Results

### Behavioral results

**Fig. 3.**
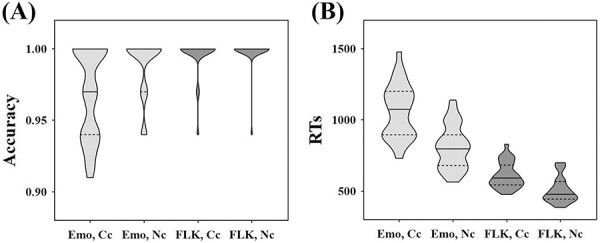
Accuracy (A) and RTs (B) for emotional conflict task and flanker task.

*Note*: Solid lines reflect medians and dotted lines represent 75% and 25% quartiles; Emo = emotional conflict task, FLK = flanker task, Nc = non-conflict condition, Cc = conflict condition.

Figure 3 displays the results of the accuracy and RT analyses. For accuracy, there was a significant main effect of task, indicating that participants’ accuracy in the flanker task (*M* = 0.997 ± 0.002) was higher than in the emotional conflict task (*M* = 0.979 ± 0.004), *F* = 20.46, *P* < 0.001. There was also a main effect of conflict condition in which accuracy was higher in the non-conflict condition (*M* = 0.993 ± 0.003) compared to the conflict condition (*M* = 0.982 ± 0.003), *F* = 8.87, *P* = 0.006. Furthermore, an interaction between task and conflict, *F* = 6.46, *P* = 0.017, revealed that the conflict effect in the emotional conflict task (*F* = 10.21, *P* = 0.004) was significantly larger than in the flanker task (*F* = 0.39, *P* = 0.537).

The RT results revealed a significant main effect of task, showing that participants responded faster in the flanker task (*M* = 558 ± 17 ms) than in the emotional conflict task (*M* = 935 ± 32 ms), *F *= 134.45, *P* < 0.001. Additionally, a main effect of conflict condition was also observed, with responses being faster in non-conflict conditions (*M* = 656 ± 19 ms) compared to conflict conditions (*M* = 837 ± 21 ms), *F *= 351.58, *P* < 0.001. Moreover, an interaction between task and conflict was found, *F *= 61.42, *P* < 0.001, suggesting that the conflict effect in the emotional conflict task (*F *= 241.48, *P* < 0.001) was significantly larger than in the flanker task (*F *= 119.78, *P* < 0.001).

### Identification of ROIs and brain networks

**Fig. 4.**
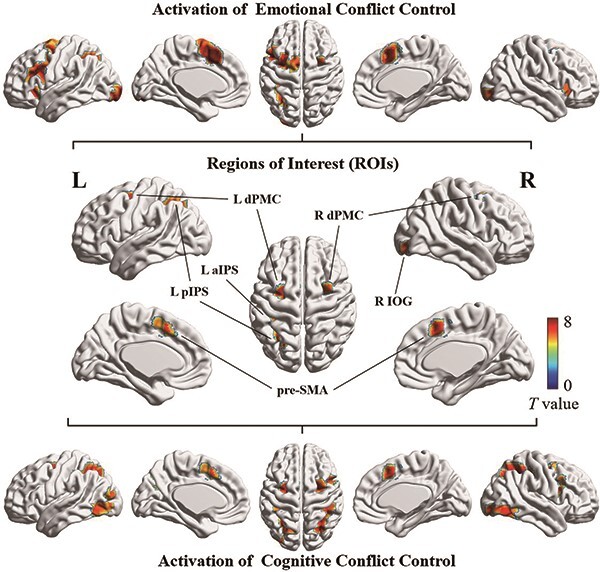
Activation maps for brain regions of emotional conflict control, cognitive conflict control and ROIs.

*Note*: Activation regions are averaged across 27 participants and mapped onto a standard ICBM152 brain template. Thresholds for the whole-brain and conjunction analyses are *p* < 0.05 (FWE corrected); only clusters with more than 10 continuous voxels are shown; L = left, R = right.

The conjunction analysis of brain activation of the two types of conflict control was performed to identify ROIs. As shown in Figure 4, thresholds for the whole-brain and conjunction analyses are *P* < 0.05, FWE corrected, and clusters with more than 10 continuous voxels above the threshold were taken as ROIs, including the pre-SMA, left and right dorsal premotor cortices (dPMCs), the left posterior intraparietal sulcus (pIPS), the left anterior IPS (aIPS), and the right inferior occipital gyrus (IOG). These six clusters were selected as ROIs and defined as nodes of the network (see details in Table 1).

**Table 1.** ROIs defined as nodes of the network

Regions	MNI (*x, y, z*)	BA	*t*	*k*
R/L pre-SMA	3 15 48	32	7.43	78
L dPMC	−27 0 51	6	6.99	47
L pIPS	−24 −60 42	7	6.82	63
R IOG	30 −90 −9	18	6.42	29
R dPMC	30 3 57	6	6.29	17
L aIPS	−36 −39 42	40	6.06	13

*Note*: L = left, R = right. MNI represents the Montreal Neurological Institute coordinates and BA represents the Brodman Area.

### Connectivity maps and hubs

Directed connectivity maps and connection strength matrixes were generated by the euSEM for emotional conflict control and cognitive conflict control. The maps revealed excellent fits to the data for all participants: in the emotional conflict task, CFI = 0.986, NNFI = 0.959, SRMR = 0.016 and RMSEA = 0.049 and in the cognitive conflict task, CFI = 0.985, NNFI = 0.957, SRMR = 0.035 and RMSEA = 0.05. According to the results of the permutation test, the two maps were significantly distinct from each other (*P* < 0.001).

**Fig. 5.**
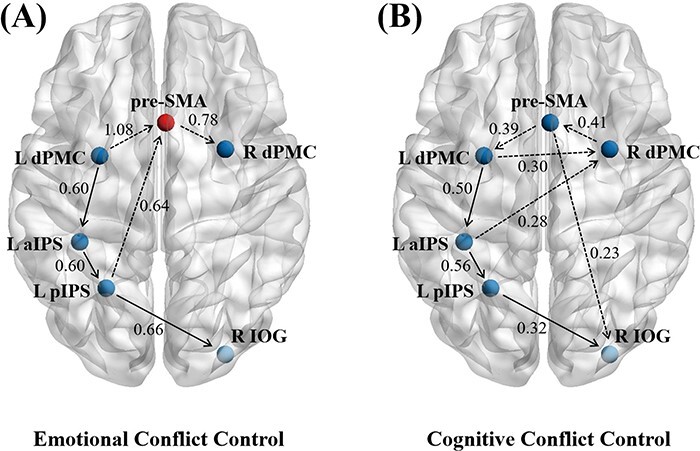
Brain connectivity maps for emotional (A) and cognitive conflict control (B).

*Note*: The standard ICBM152 brain template was used for brain connectivity maps. All lines represent contemporaneous relations after controlling for time-lagged relations and the modulating effects. Solid lines indicate shared connections, and dashed lines refer to specific connections. Numbers represent connection strengths. The pre-SMA node was the core of the Emotional Conflict Control network. L = left, R = right.

The connectivity map of the emotional conflict control revealed a well-interconnected network and the optimal core–periphery analysis showed that the pre-SMA was the core of the network (*Q* = 0.571) (see Figure 5A). The pre-SMA received influence from the left dPMC [connection strength (*β*) = 1.08] and projected information to the right dPMC (*β* = 0.78). Moreover, neural signals from the left dPMC also flowed along the left aIPS (*β* = 0.60) and the left pIPS (*β* = 0.60) to the right IOG (*β* = 0.66). The left pIPS then sent signals to the pre-SMA (*β* = 0.64) as well.

**Fig. 6.**
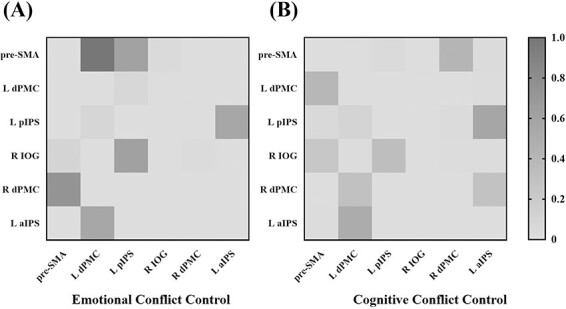
Connection strength matrixes of emotional (A) and cognitive conflict control (B).

*Note*: L = left, R = right.

The connectivity map of cognitive conflict control showed both similar and distinct patterns as the connective map of emotional conflict control (see Figure 5B). The similarity was that neural signals from the left dPMC flowed along the left aIPS (*β* = 0.50) and the left pIPS (*β* = 0.56) to the right IOG (*β* = 0.32). Differentially, no core was found in the cognitive conflict control network, and information flowed along the pre-SMA, the left dPMC, and the right dPMC, forming neural circuits: from the pre-SMA to the left dPMC (*β* = 0.39), from the left dPMC to the right dPMC (*β* = 0.30), and from the right dPMC to the pre-SMA (*β* = 0.41). In addition, the pre-SMA exerted influence on the right IOG (*β* = 0.23), while the left aIPS had an effect on the right dPMC (*β* = 0.28). The connection strength matrixes of emotional conflict control and cognitive conflict control were shown in Figure 6.

### Shared connections

**Fig. 7.**
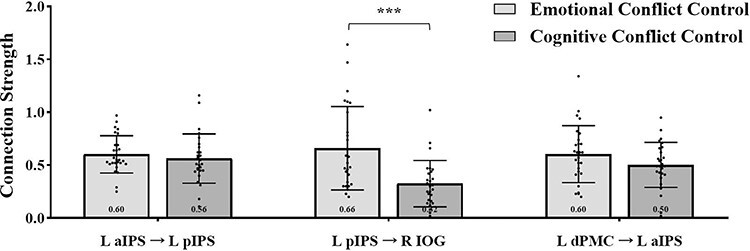
Strength differences between shared connections for emotional and cognitive conflict control.

*Note*: L = left, R = right. ***P < 0.001.

The two maps shared three connections: left dPMC → left aIPS → left pIPS → right IOG. As shown in Figure 7, the strength of the shared connection from the left dPMC to the left aIPS and from the left aIPS to the left pIPS did not show a significant difference, *P*s > 0.15. The strength of the shared connection from the left pIPS to the right IOG was significantly distinct, such that stronger connections were found in the emotional conflict control map compared to the cognitive conflict control map, *z* = 3.676, *P* < 0.001.

## Discussion

In the present study, we used fMRI to examine the neural basis of emotional conflict control and cognitive conflict control. We found that the multitasking-related activity in the pre-SMA, bilateral dPMCs, the left pIPS, the left aIPS, and the right IOG during both types of conflict control represented common subprocesses in controlling the efficient performance of the two tasks. Using euSEM, we explored the connectivity patterns of these critical brain regions separately for two conflict controls. The results showed connectivity maps for emotional conflict control and cognitive conflict control and both showed excellent interconnectivity in the domain-general conflict control network (i.e. the overlapping regions of the two). The results also revealed that emotional conflict control reconfigured the brain network of domain-general conflict control by modulating the connectivity pattern. In the next subsections, we will elaborate on this reconfiguration and the similarities and differences of connectivity patterns between emotional and cognitive conflict control.

The results showed that emotional and cognitive conflict control shared connectivity such that the left dPMC flowed along the left aIPS to the left pIPS. The left dPMC cluster obtained in our conjunction analysis overlapped with the presumed location of the human frontal eye field (FEF; Paus, 1996; Zu Eulenburg *et al*., 2012). The FEF is thought to facilitate visual target detection (Grosbras and Paus, 2003) by biasing perception through attentional top-down signals (Corbetta and Shulman, 2002; Langner *et al*., 2012). The IPS, between the inferior parietal lobule and superior parietal lobule, is a key region for attention control (Geng and Mangun, 2009; Gillebert *et al*., 2011) and has also been reported to be involved in conflict processing (Friedman-Hill *et al*., 2003; Luks *et al*., 2007). Additionally, the aIPS is suggested to be related to top-down reorienting of attention when responding to spatially incongruent targets (Cieslik *et al*., 2010). The pIPS has been found to facilitate target discrimination after a conflict trial by directing attention to features of task-relevant stimuli (Soutschek *et al*., 2013). The causal role of the pIPS in target discrimination has been supported in a study employing both fMRI and transcranial magnetic stimulation (TMS) (Capotosto *et al*., 2013).

The IPS as a neural mechanism for attentional control has also been reported in conflict studies. For instance, Luks *et al*. (2007) found that the IPS was related to preparatory attentional allocation during conflict control, as shown by increased IPS activity during the informative cue period and subsequent decreased IPS activity during conflict-related target processing. In the present study, the effective connectivity of these regions in both types of conflict control may represent the mechanism of attentional control. That is, conflict conditions served as salient events which the left dPMC detected, and then the incongruent signal was transmitted to the left aIPS for attention reorienting, where the left pIPS directed attention to features of task-related stimuli to facilitate target recognition. This result is in line with previous research that considered FEF and IPS to be part of the dorsal attention network (DAN), a network that facilitates top-down control of attention for both voluntary and goal-directed behavior (Corbetta and Shulman, 2002; Corbetta *et al*., 2008).

In addition to the brain regions discussed earlier, in the present study, attentional control was also related to the right IOG, as shown by the effective connectivity from the left pIPS to the right IOG in both emotional and cognitive conflict control. The IOG has been found in many studies examining conflict resolution (Chechko *et al*., 2012; Xu *et al*., 2016) but was usually seen as an independent visual network (Yeo *et al*., 2011). The IOG also plays a key role in attentional modulations (Grosbras *et al*., 2005; Hietanen *et al*., 2006), and the occipital regions are sometimes attributed to the DAN (Benedek *et al*., 2016). However, our results showed that the connection strength from the left pIPS to the right IOG was significantly stronger in emotional conflict control than in cognitive conflict control. It is possible that either the more complex stimuli of emotional conflict have higher demands for attention control or that they may be related to the modulation of emotional involvement on attention control. Studies have shown that individuals more readily pay attention to emotional than neutral stimuli (Richards and Blanchette, 2004) and that emotional material biases attentional resource deployment, producing an exacerbated attentional blink (Yiend, 2010).

In the emotional conflict control brain network, the pre-SMA was found to be the core of the network, received influence from the attention network (i.e. the left dPMC and the left pIPS), and then projected information to the right dPMC. Several meta-analyses have shown that the pre-SMA, dorsal to the anterior cingulate cortex, is a critical brain region involved in conflict control across various conflict paradigms (Xu *et al*., 2016). This region is considered to be associated with conflict monitoring, in which signals are sent to other regions when a conflict is identified (Garavan *et al*., 2003; Iannaccone *et al*., 2015). The dPMC, adjacent to the posterior primary motor cortex, is also involved in conflict control. TMS studies have reported that stimulation of dPMCs promotes response selection, an important part of conflict control (Koski *et al*., 2005; Chambers *et al*., 2007;Duque *et al*., 2012). For instance, Chambers *et al*. (2007) reported a functional separation of dPMC and prefrontal cortex, in that the stimulation of the right dPMC facilitated execution but had no effect on inhibition and the stimulation of the right PFC impaired inhibition. In the present study, for emotional conflict control, the pre-SMA may be responsible for the detection of conflicting information, such that, under the direction of the attention network, it monitored interference that was triggered by conflicting emotional valence and then signaled other areas, such as the right dPMC, to implement subsequent conflict control and to achieve the response selection (Chambers *et al*., 2007).

Moreover, in the emotional conflict control brain network, the highly efficient connection between the prefrontal and parietal regions was consistent with previous studies in which the network consisting of prefrontal and parietal regions was often activated in emotional control tasks in which participants are explicitly asked to regulate their emotions (Buhle *et al*., 2014; Kohn *et al*., 2014). Emotional regulation refers to intentionally generating, enhancing, reducing or stopping a given emotion (Langner *et al*., 2018). Emotional conflict control in the present study can also be viewed as a form of emotional regulation (i.e. participants under the conflict condition were asked to change words’ emotional valence to the opposite valence), thus requiring a favorable interaction between the frontal and parietal cortices to facilitate the regulation of emotion. The frontoparietal network consisting of the IPS and the prefrontal cortex have been demonstrated in neurocognitive models as a crucial element in the perception of emotional stimuli (Pourtois *et al*., 2013; Fan *et al*., 2018).

For the cognitive conflict control brain network, the right dPMC received information from the attention network (i.e. the left dPMC and the left aIPS), and then information flowed along the pre-SMA, the left dPMC, and back to the right dPMC. Studies have shown that the dPMC is associated with the selection of correct responses in conflict control (Koski *et al*., 2005; Chambers *et al*., 2007; Duque *et al*., 2012). The prominent contribution of bilateral dPMCs in the current study may have emerged because the flanker task required a greater demand for resources that support response selection and action execution, compared with conflict monitoring or interference suppression. Consistent with this finding, O’Shea *et al*. demonstrated that stimulation of the left dPMC led to adaptive reorganization of the right dPMC to mediate response selection.

Interestingly, neural signals seemed to form a premotor circuit along the right dPMC, the pre-SMA, the left dPMC, and back to the right dPMC. This implies that the pre-SMA was more involved in response selection and action execution with bilateral dPMCs during cognitive conflict control. This speculation accords with previous studies in which the pre-SMA, located between the prefrontal and motor systems, was hypothesized to participate in higher-level functions related to executive control of motor skills, such as resolving conflicting responses (Nachev *et al*., 2007) and selecting correct responses (van Gaal *et al*., 2011). For instance, Nachev *et al*. (2007) reported that patients with lesions in the pre-SMA displayed an impairment for inhibiting competing motor plans in conflict situations. Moreover, gray matter density in the pre-SMA has been found to be positively correlated with the ability to voluntarily select the correct action when resolving conflicting responses (van Gaal *et al*., 2011).

Finally, to examine how emotional conflict control reconfigures the domain-general conflict control brain network, we explored the neural separation of two conflict controls at the level of specific interaction patterns in brain regions, which adds to the exploratory nature of this study. Nonetheless, some limitations of our study merit mention. First, the roles of various brain regions discussed earlier are assumptions based on previous studies and focus only on the overlaps, and ignoring regions relevant to a specific task may cause us to lose some information. The results may be different when considering the full network. Thus, the objective of our study was not to examine the specific role played by each brain region in different conflict controls, but to underscore that even the domain-general conflict control network involved in different conflict controls at the same time is regulated by conflict types and shows unique connectivity patterns. Second, the emotional conflict task in the present study only contained results for positive words. It should be pointed out that conflict control for negative words may also display specific patterns of brain network connectivity. Third, the differences in task relevance of the distracting stimuli in the two tasks (i.e. the distracting stimuli in the emotional conflict task were task relevant, whereas the distracting stimuli in the flanker task were task irrelevant) may also affect the neural separation of the identified connectivity. Future work should consider other tasks that can also address the objectives of this study (i.e. the contextualization of emotional conflict control and the representation of the cognitive conflict control) while ensuring a balance between task-relevant and task-irrelevant stimuli across experiments. This issue and the other limitations mentioned earlier provide a foundation for further exploration in future studies.

## Conclusion

The present study examined overlapping brain networks involved in emotional conflict control and cognitive conflict control and separately explored their effective connectivity patterns using euSEM. Specifically, the findings showed that the distributed frontoparietal and occipital network that was activated across tasks worked together in emotional conflict control and cognitive conflict control to support the different cognitive demands of conflict control. The present study has also revealed that emotional conflict control reconfigures the brain network of domain-general conflict control in a connective way. This reconfiguration was reflected in the fact that emotional conflict control recruited different communication and strengthened connectivity among the domain-general conflict control network. Together, these findings offer novel evidence for the neural relationship between emotional conflict control and cognitive conflict control and provide an empirical-based elaboration on the brain network underpinning emotional conflict control and how it reconfigures the domain-general conflict control network in connective ways.

## Supplementary Material

nsae001_Supp

## Data Availability

The datasets generated and analyzed in this study are available in the Open Share Freedom (OSF) repository: Liu, H. (4 February 2023). Emotional Conflict Control and Cognitive Conflict Control. Retrieved from osf.io/5ecwn. This dataset has also been used in our previous research *(Guo et al., 2023)*.

## Footnotes

1 For the ANOVA test, Cohen suggested the effect sizes of ‘small,’ ‘medium’ and ‘large’ as 0.1, 0.25 and 0.4, respectively (see Kang, 2021).

2 The 36 neutral words included 18 neutral words that were artificially characterized as positive words and 18 neutral words that were artificially characterized as negative words. Participants were familiar with them before the formal experiment. For the neutral words, they performed the same task with emotional words, but no emotion was actually involved. Thus, it was considered a control condition and not included in the analyses.

3 This is consistent in empirical and simulated studies examining the likelihood of detecting a true effect should it exist in a given sample (Hillary *et al*., 2011; Gates and Molenaar, 2012).
