# Supplementary material for: Effects of contextualized emotional conflict control on domain-general conflict control: fMRI evidence of neural network reconfiguration
Source: Soc Cogn Affect Neurosci. 2024 Jan 4;19(1):nsae001. doi: 10.1093/scan/nsae001 (PMC10868129; doi:10.1093/scan/nsae001)
Supplement: nsae001_Supp [file nsae001_supp.zip › scan-23-072-File003.docx]

**Target Words (and English Translations) in the Emotional Conflict Task**

| **Positive** | | **Negative** | |
| --- | --- | --- | --- |
| **Emotional** | **Neutral** | **Emotional** | **Neutral** |
| 快乐 (happy) | 总是 (always) | 悲伤 (sad) | 从不 (never) |
| 兴奋 (excited) | 巨大 (big) | 困倦 (tired) | 细小 (small) |
| 喜欢 (like) | 收到 (receive) | 耻辱 (shame) | 送出 (send) |
| 愉快 (merry) | 快速 (quick) | 恼火 (anger) | 慢速 (slow) |
| 轻松 (easy) | 飞快 (quickly) | 痛苦 (painful) | 缓慢 (slowly) |
| 幸福 (happiness) | 长的 (long) | 紧张 (nervous) | 短的 (short) |
| 自豪 (pride) | 买入 (buy) | 憎恨 (hate) | 卖出 (sell) |
| 高兴 (glad) | 借入 (borrow) | 焦虑 (anxiety) | 借出 (lend) |
| 开心 (rejoice) | 许多 (many) | 害怕 (afraid) | 很少 (few) |
| 幸运 (lucky) | 提高 (increase) | 心烦 (upset) | 降低 (decrease) |
| 满足 (satisfied) | 出现 (appear) | 内疚 (guilty) | 消失 (disappear) |
| 愉悦 (cheerful) | 上升 (ascend) | 无聊 (boring) | 下降 (descend) |
| 欢乐 (joy) | 继续 (continue) | 嫉妒 (jealous) | 暂停 (pause) |
| 有趣 (funny) | 宽阔 (wide) | 糟糕 (terrible) | 狭窄 (narrow) |
| 享受 (enjoy) | 多数 (large) | 尴尬 (awkward) | 少数 (little) |
| 喜爱 (love) | 高的 (high) | 愚蠢 (foolish) | 低的 (low) |
| 成功 (success) | 众多 (numerous) | 恐惧 (fear) | 稀少 (rare) |
| 温暖 (warm) | 增加 (add) | 厌恶 (disgusted) | 减少 (reduce) |

**Table S1**

*Conflict Effects of Positive Words Involved in the Emotional Conflict Task*

| Regions | *k* | *MNI (x,y,z)* | *BA* | *t* |
| --- | --- | --- | --- | --- |
| L inferior occipital gyrus | 104 | -24 -93 -9 | 18 | 10.12 |
| R/L pre-supplementary motor area | 176 | -3 15 48 | 32 | 9.95 |
| R insula | 24 | 36 21 3 | 48 | 8.94 |
| L dorsal premotor cortex | 50 | -30 0 54 | 6 | 8.47 |
| L superior parietal lobule | 73 | -27 -63 48 | 7 | 7.95 |
| L lateral prefrontal cortex | 107 | -42 6 27 | 44 | 7.87 |

*Notes.* FWE Corrected, *p_voxel_* < .05, *p_cluster_* < .001; Cluster threshold of *k* > 20; Cluster threshold of *k* > 20. L = Left, R= Right.

**Table S2**

*Conflict Effects Involved in the Flanker Task*

| Regions | *k* | *MNI (x,y,z)* | *BA* | *t* |
| --- | --- | --- | --- | --- |
| R inferior occipital gyrus | 89 | 57 -63 6 | 37 | 5.49 |
| R lateral prefrontal cortex | 112 | 45 42 15 | 45/46 | 5.32 |
| L inferior occipital gyrus | 61 | -51 -81 6 | 19 | 4.87 |
| R supramarginal gyrus | 84 | 54 -33 45 | 40 | 4.81 |

*Notes.* *p*_voxel_ < .05, *p*_cluster_ < .001, uncorrected; Cluster threshold of *k* > 20. L = Left, R= Right.
